# Work–Family Boundary Fit and Employee Well-Being: The Mediating Role of Work–Family Conflict

**DOI:** 10.3390/bs15081122

**Published:** 2025-08-19

**Authors:** Ying Meng, Hongying Li, Yong Qu, Guilan Yu

**Affiliations:** 1 School of Business Management, Jilin University of Finance and Economics, Changchun 130117, China; mengying@jlufe.edu.cn (Y.M.); 114045@jlufe.edu.cn (H.L.); 2 School of Business and Management, Jilin University, Changchun 130012, China; yugl@jlu.edu.cn

**Keywords:** boundary management, boundary preference, boundary enactment, work–family conflict, employee well-being

## Abstract

This study explores how the consistency between work–family boundary preferences and boundary enactment affects employee well-being by applying polynomial regression and response surface analysis. Using two-wave data from 420 employees in consulting firms in China, recruited via snowball sampling, bootstrapping was conducted to examine whether reduced work–family conflict mediates this relationship. The results show that consistency between preferences and enactment is positively related to job satisfaction and depression–enthusiasm well-being but has no significant effect on anxiety–comfort well-being. Anxiety–comfort and depression–enthusiasm well-being are higher when both preferences and enactment are high compared to when both are low. Work–family conflict plays a mediating role in this relationship. This research adds to the boundary management literature by highlighting the importance of aligning work–family boundary preferences with enactment. The findings suggest that organizations can improve employee well-being by supporting boundary management practices that match individual preferences.

## 1. Introduction

The COVID-19 pandemic has profoundly transformed work arrangements worldwide, accelerating the adoption of remote work and digital collaboration tools. This shift has blurred traditional boundaries between work and family roles, creating new challenges for individuals striving to balance these domains. While some individuals prefer a segmented boundary approach that strictly separates work and family, others favor integration, blending the two spheres to leverage flexibility and resource availability. Despite organizations increasingly endorsing flexible and remote work policies that encourage integration, there remains considerable variation in employees’ boundary preferences and enactments. Understanding how these boundary dynamics affect employee well-being is critical, particularly in the post-pandemic context marked by evolving occupational health concerns.

Previous studies have extensively explored the relationship between boundary management and well-being from both individual perspectives (e.g., strategies or preferences for managing work and family roles) and organizational perspectives (e.g., policies, practices, or environmental constraints). However, several key gaps remain to be addressed. First, much of the prior research has focused on individual boundary preferences and the influence of organizational environments (Foucreault et al., 2018; Fu et al., 2021; Lin et al., 2022; Zhang et al., 2024), while paying relatively less attention to the actual enactment of boundaries by individuals. Boundary preferences differ from actual boundary enactment, and the latter is a critical aspect of boundary management that cannot be overlooked. Second, while some scholars have begun investigating the interaction effects of variables, such as the alignment between individual preferences and organizational environments from a person–environment fit perspective, and their influence on well-being and work–family domain outcomes (Amirkamali et al., 2024; Kreiner, 2006; H. Ma et al., 2014; L. Ma & Ma, 2019; L. Ma & Xu, 2011; Rothbard et al., 2005), the interconnection or alignment between boundary preferences and actual boundary enactment has been largely neglected. Such alignment may offer stronger explanatory power for certain variables in the work–family domain (Ammons, 2013). Meeting expectations regarding work–family boundaries could be a significant source of well-being. Third, although Allen et al. (2014) emphasized the need to examine the consistency between boundary management preferences and actual boundary enactment, few scholars have explored this consistency (Michel et al., 2022; Urbanaviciute et al., 2023). To our knowledge, no prior empirical study has used polynomial regression to test the relationship between the alignment of boundary preferences and enactment with well-being, and no studies have investigated the underlying mechanisms explaining this relationship either.

To address these gaps, this study aims to investigate whether and how the fit between employees’ work–family boundary preference and their boundary enactment affects their well-being, with a particular focus on the mediating role of work–family conflict. The focus of this research is on employee well-being, which includes job satisfaction (employees’ evaluation of their work) and job-related affective well-being, which is defined by Warr (1990)’s circumplex model, encompassing the two dimensions of anxiety–comfort and depression–enthusiasm. We adopt a person-centered approach integrating boundary theory and role theory to explore this relationship, hypothesizing that work–family conflict mediates the effect of preference–enactment alignment on well-being. This choice is grounded in role theory (Kahn et al., 1964), which emphasizes that work and family are distinct roles that often compete for limited time and energy, leading to inter-role conflict. When employees are unable to manage boundaries in accordance with their preferences, the resulting misfit can heighten psychological strain and role ambiguity, both of which are primary antecedents of work–family conflict (Greenhaus & Beutell, 1985). Thus, work–family conflict serves as a key explanatory process linking boundary preference–enactment (mis)alignment to employee well-being.

Using polynomial regression and response surface analysis, this study makes theoretical contributions in the following three aspects: First, departing from the person–environment fit perspective, this research adopts an employee-centered approach to explore the mechanisms by which the alignment between boundary preferences and boundary enactment influences well-being. This perspective contributes to a deeper theoretical understanding of the relationship between boundary management and well-being. Second, we integrate boundary theory with role theory, theoretically linking work–family boundary preferences and the alignment between boundary preferences and enactment to well-being, with work–family conflict proposed as a mediating mechanism. This integration contributes to advancing theoretical unification in this domain. Third, by identifying work–family conflict as the mediating variable through which the alignment between boundary preferences and enactment affects well-being, this study delineates the underlying mechanisms of their relationship, offering robust explanations for the connection between these constructs.

## 2. Literature Review and Hypotheses

### 2.1. Boundary Theory and Boundary Management

Boundary theory serves as the foundation for research on boundary management, proposing that individuals create, maintain, and modify boundaries to simplify and organize their surrounding world (Ashforth et al., 2000). Work–family boundaries delineate work and family as two distinct domains (Ashforth et al., 2000; Nippert-Eng, 1996). Strategies for managing work–family boundaries exist on a segmentation–integration continuum (Allen & Finkelstein, 2014; Bulger et al., 2007), which refers to the degree to which one domain (work/family) is kept separate from the other (family/work) (Kreiner, 2006).

Under complete segmentation, the boundaries between roles are clear, with no conceptual, physical, or temporal overlap. For example, individuals may avoid responding to work-related calls after work hours. Under complete integration, work and family overlap, allowing activities, objects, people, ideas, and emotions from one domain to enter the other (Nippert-Eng, 1996). Most people adopt boundary management strategies that fall between these two extremes.

Boundary preference and boundary enactment are key concepts in the domain of boundary management. Boundary preference is an individual difference variable that reflects the extent to which a person prefers work and family to be segmented or integrated (Ammons, 2013; Kreiner, 2006). Boundary enactment represents the actual level of segmentation or integration established to fulfill work and family roles (Allen et al., 2014; Amirkamali et al., 2024).

Since work–family boundaries exist on a segmentation–integration continuum, this paper uses the general terms boundary preference and boundary enactment when discussing concepts broadly. However, when examining the relationships between boundaries and other constructs, the terms high/low-boundary segmentation preference and high/low-boundary segmentation enactment are used to indicate the directionality of the relationship.

Additionally, the degree of segmentation or integration may differ for work-to-family boundaries compared to family-to-work boundaries (Amirkamali et al., 2024; Matthews & Barnes Farrell, 2010). This study focuses on boundary management issues in the work-to-family direction.

### 2.2. Pairing of Boundary Preference and Actual Boundary Enactment

**Figure 1:**
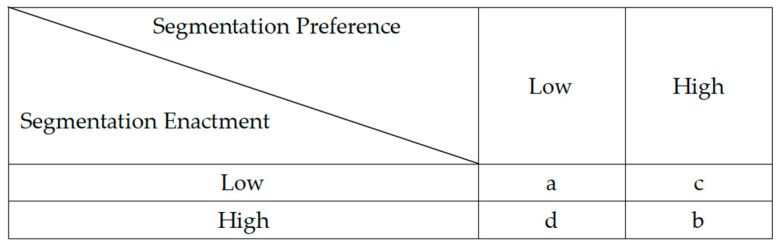
Segmentation preference and segmentation enactment pairing scenarios.

Based on employees’ boundary segmentation preference and boundary segmentation enactment, four pairing scenarios can be formed, as shown in Figure 1. Among these, scenarios a and b represent alignment between boundary preference and boundary enactment, while scenarios c and d indicate misalignment between preference and enactment.

### 2.3. The Relationship Between the Consistency of Boundary Preference and Boundary Enactment and Well-Being

Employee well-being is often treated as a multi-dimensional construct (Diener, 1984), encompassing both cognitive (e.g., job satisfaction) and affective components (e.g., anxiety–comfort and depression–enthusiasm). In this study, we conceptualize three dimensions widely used in previous work–family domain studies (Mielniczuk & Łaguna, 2018; Wood et al., 2018) to represent distinct but interrelated aspects of job-related well-being.

From a self-determination theory perspective, well-being arises when individuals’ basic psychological needs, including autonomy, competence, and relatedness, are met (Ryan & Deci, 2000). In the context of boundary management, we argue that boundary preferences reflect an individual’s autonomy needs in managing work and nonwork roles. That is, people differ in the degree to which they prefer to segment or integrate work and family domains. When employees are able to enact boundaries that align with these preferences, their autonomy need is satisfied, leading to higher well-being. This process reflects a form of autonomy support, defined as experiencing freedom and endorsement of one’s choices across social contexts (Deci et al., 1989).

Boundary theory (Clark, 2000) complements this perspective by positing that boundary management occurs through interaction between individuals and role stakeholders, such as supervisors or family members, who function as boundary keepers. When alignment occurs between preference and enactment, it often implies that others are allowing the individual to structure boundaries in ways that suit them—thus reinforcing autonomy support across domains. Previous research has shown that such perceived autonomy support is a reliable predictor of workplace satisfaction and affective well-being (Patrick et al., 2007). Therefore, when boundary preferences align with boundary enactment, employees are likely to experience higher levels of well-being (Michel et al., 2022).

When individuals’ segmentation preferences exceed their segmentation enactment, it indicates that their actual boundaries are more integrated than their preferences. Integrated boundaries require frequent transitions between roles, potentially leading to role ambiguity and reduced well-being (Carvalho et al., 2025; Desrochers et al., 2005; Rothbard et al., 2005). Conversely, when individuals’ segmentation preferences are lower than their actual segmentation enactment, it suggests that their boundaries are more segmented than desired. Excessive segmentation may hinder role transitions, creating challenges for individuals. Based on this, we propose the following hypotheses:

**H1a.**  *The alignment between boundary preferences and boundary enactment is positively associated with job satisfaction.*

**H1b.**  *The alignment between boundary preferences and boundary enactment is positively associated with job-related anxiety–comfort.*

**H1c.**  *The alignment between boundary preferences and boundary enactment is positively associated with job-related depression–enthusiasm.*

Job satisfaction reflects a general cognitive evaluation of one’s job and is influenced by the extent to which work experiences align with personal values and preferences (Judge et al., 2006). When boundary preferences and boundary enactment are aligned, employees perceive a sense of congruence between what they desire and what they experience, which supports psychological contract fulfillment and enhances job attitudes (Edwards & Rothbard, 2000). This positive evaluative process can occur regardless of whether the alignment reflects segmentation or integration preferences, as both forms can be equally desirable depending on individual preference. For example, when both segmentation preference and enactment are high, employees can effectively protect their nonwork roles, while when both are low, they may experience greater role blending, which is consistent with their preference for integration.

However, affective well-being, such as job-related anxiety–comfort or depression–enthusiasm, depends more on how work–family boundaries affect emotional strain. Drawing on role scarcity theory (Kahn et al., 1964), segmented boundaries allow individuals to conserve and allocate finite resources like time and energy more efficiently, reducing inter-role interference. High segmentation reduces cross-role spillover, thus minimizing confusion, emotional overload, and stress (Ashforth et al., 2000; Edwards & Rothbard, 2000). In contrast, low levels of boundary segmentation are associated with role ambiguity and conflict, which can lead to emotional experiences such as exhaustion and anxiety (Wood et al., 2018). Therefore, employees who both prefer and enact high segmentation may experience lower anxiety and higher emotional energy compared to those in a low preference–low enactment state. Based on these considerations, we propose the following hypotheses:

Job satisfaction represents an overall evaluation of one’s work. The alignment between boundary preferences and boundary enactment suggests that work meets an individual’s expectations to some extent, leading to a higher overall evaluation of the job, regardless of whether the alignment involves segmentation or integration. On one hand, when both segmentation preferences and segmentation enactment are high, the job helps individuals achieve their desire to separate work from the family domain. Conversely, when both preferences and enactment lean toward integration, the job facilitates the individual’s desire to blend work and family.

However, job-related affective well-being differs. According to the scarcity perspective of role theory (Kahn et al., 1964), time and energy are limited resources that individuals allocate across various roles, making role segmentation necessary. Role segmentation allows individuals to clearly and distinctly fulfill the roles associated with work and family, reducing interference between these roles and mitigating role conflict and ambiguity (Ashforth et al., 2000; Nippert-Eng, 1996). Clear work–family boundaries promote role separation between work and family, enabling individuals to effectively manage both domains and minimize conflicts between work and family roles (Edwards & Rothbard, 2000). Based on these arguments, we propose the following hypothesis:

**H2a.**  *Job satisfaction does not significantly differ when both preferences and enactment are high compared to when both are low.*

**H2b.**  *Anxiety–comfort well-being is higher when both preferences and enactment are high compared to when both are low.*

**H2c.**  *Depression–enthusiasm well-being is higher when both preferences and enactment are high compared to when both are low.*

### 2.4. The Mediating Role of Work–Family Conflict

Work–family conflict is a concept developed based on role theory. Greenhaus and Beutell (1985) defined work–family conflict as a specific form of inter-role conflict which arises when work-related time demands prevent individuals from meeting the needs of their family roles; when work stress results in tension symptoms such as fatigue and irritability, impairing performance in family roles; or when expected behaviors in the work role (e.g., detachment) conflict with expected behaviors in the family role (e.g., warmth).

Work–family conflict (WFC) refers to inter-role tension in which pressures from work and family roles are mutually incompatible (Greenhaus & Beutell, 1985). The boundary fit literature suggests that alignment between boundary preferences and enactment reduces role conflict and ambiguity by allowing individuals to manage domains in ways that align with their internal values and expectations (Kossek et al., 2011; Mellner et al., 2021). Such fit provides predictability and control over role transitions, which are essential for reducing role strain (Abdou et al., 2024).

From a psychological perspective, alignment increases a sense of autonomy and reduces cognitive dissonance (Ryan & Deci, 2000). This improved congruence reduces internal friction between ideal and actual states, making domain management more fluent and less conflict-laden. Conversely, being forced to integrate when one prefers segmentation can increase emotional strain and WFC by undermining personal control and increasing unpredictability (Chuang et al., 2025).

Furthermore, consistent with conservation of resources theory (Hobfoll, 1989), reduced WFC preserves emotional and cognitive resources, thereby improving well-being outcomes (Bölingen et al., 2023; Powell & Greenhaus, 2010; Weinert et al., 2024; Wood et al., 2018). When WFC is minimized, employees experience fewer stressors that impair affective states, such as anxiety or depression, and are more likely to evaluate their jobs positively.

Based on this, we make the following hypotheses:

**H3a.**  *Work–family conflict mediates the positive relationship between boundary preference–boundary enactment alignment and job satisfaction.*

**H3b.**  *Work–family conflict mediates the positive relationship between boundary preference–boundary enactment alignment and job-related anxiety–comfort well-being.*

**H3c.**  *Work–family conflict mediates the positive relationship between boundary preference–boundary enactment alignment and job-related depression–enthusiasm well-being.*

## 3. Methods

### 3.1. Procedure and Sample

Participants were recruited by sending invitation emails to MBA students from a business school working in consulting companies and using snowball sampling (Goodman, 1961). Each individual in the sample was working in a consulting company in China and born after 1970. The reason for selecting consulting company employees as the sample is that they typically have to communicate with the customer and provide solutions regarding the consulting program provided to the customer at any time. These employees are more likely to work in nonwork places and/or complete nonwork hours. Therefore, their problems related to work–family boundary management may be more pronounced.

Participants completed two online surveys with a one-week interval between July and August 2023. The surveys were administered in Chinese, the participants’ native language. The original English scales were translated and back-translated following standard procedures to ensure conceptual equivalence, thus eliminating potential language barriers. The first survey asked questions about boundary preference, work–nonwork boundary enactment, life role priority, and demographics, as well as asking for the last 6 digits of the participant’s cell phone number. The second survey measured work–nonwork conflict and employee well-being, also asking for the last 6 digits of their cell phone number. We used the last 6 digits of each participant’s cell phone number to match the surveys collected at the two separate times.

Overall, 720 participants complete the survey at time 1, and 420 participants complete the survey at time 2. The sample consisted of 37.1% male participants and 62.9% female participants. In total, 52.4% were born between 1990 and 1999, 42.9% were born between 1980 and 1989, and 4.8% were born between 1970 and 1979. Additionally, 22.4% of the respondents were single (including separated, divorced, and widowed individuals), 77.6% lived with a spouse or partner, and 57.9% had children living in their home. Moreover, 7.4% of the sample belonged to top management positions, 54.8% of all employees held a supervisory position, 34.3% of the were employees without a supervisory function, and 3.6% of the employees were trainees/interns. On average, they worked 45.19 h in the week, with a standard deviation of 8.93.

### 3.2. Measures

#### 3.2.1. Work–Family Boundary Preference

We measured work–family boundary preference using the 4-item scale developed by Kreiner (2006). This scale is grounded in boundary theory and has been widely validated to assess individuals’ segmentation–integration preference. Sample items included ‘I prefer to keep work life at work’ and ‘I like to be able to leave work behind when I go home’. Cronbach’s alpha was 0.703.

#### 3.2.2. Work–Family Boundary Enactment

We measured work–family boundary enactment using the 5-item scale developed by Wepfer et al. (2017). This scale captures actual behavioral boundary management’s consistency with boundary theory. Respondents were asked to indicate where they place themselves on a 5-point Likert scale between the polar statements of each item. Sample items included ‘I often take work home–never take work home’ and ‘I often leave my workplace late–I always leave my workplace on time’. Cronbach’s alpha was 0.741.

#### 3.2.3. Work–Family Conflict

We used a 5-item scale developed by Netemeyer et al. (1996) to measure work demands interfering with family. This scale is a classic measure of role interference commonly used in work–family research. Sample items included ‘The demands of my work interfere with my family and personal life’. Cronbach’s alpha was 0.837.

#### 3.2.4. Employee Well-Being

Employee well-being is measured through multi-dimensional scales. Job satisfaction reflects cognitive evaluation of work, while job-related anxiety–contentment and job-related depression–enthusiasm scales (Warr, 1990) capture affective well-being, consistent with theoretical constructs of subjective well-being.

Job satisfaction was measured by asking respondents to indicate their level of agreement in a scale, ranging from 1 (strongly disagree) to 5 (strongly agree), developed by Hackman and Lawler (1971) with 3 items. Sample items included ‘Generally speaking, I am very satisfied with this job’ and ‘Generally speaking, I am very satisfied with the kind of work I do on my job’. Cronbach’s alpha was 0.768.

Job-related anxiety–contentment was measured by the Warr (1990) anxiety–contentment scale, which is based on answers to the question, ‘Thinking of the past few weeks, how much of the time has your job made you feel…?’, for three negative items—tense, worried, and uneasy. The survey adopted a five-point scale: ‘all of the time’, ‘most of the time’, ‘some of the time’, ‘occasionally’, or ‘never’. Cronbach’s alpha was 0.735.

Job-related depression–enthusiasm was measured by the Warr (1990) depression–enthusiasm scale, considering items such as depressed, gloomy, and miserable. The survey adopted a five-point scale: ‘all of the time’, ‘most of the time’, ‘some of the time’, ‘occasionally’, or ‘never’. Cronbach’s alpha was 0.768.

#### 3.2.5. Control Variables

Control Variables were determined based on prior boundary research (Foucreault et al., 2018; Powell & Greenhaus, 2010; Wepfer et al., 2017). We considered gender (1 = male; 2 = female), age (1 = 90 s; 2 = 80 s; 3 = 70 s), marital status (1 = single; 2 = living with a partner without marriage; 3 = married), whether an individual had children living in their home (1 = yes; 2 = no), job status (1 = trainee/intern; 2 = employees without supervisory function; 3 = junior and middle-level managers; 4 = senior and top managers), hours worked (assessed as a continuous variable), and organizational tenure; the latter was based on number of years worked for current organization (assessed as a continuous variable), and life role priority served as a control variable.

Life role priority was assessed by asking for responses to two statements (Allen & Finkelstein, 2014)—‘How often do you feel that you put your job before your personal or family life?’ and ‘How often do you feel that you put your personal or family life before your job?’—on a 5-point scale from 1 (never) to 5 (very often). The second responses were reverse-coded so that higher scores represented greater work priority.

### 3.3. Analyses

We used polynomial regression with response surface analysis (Edwards & Parry, 1993; Shanock et al., 2010) to test Hypothesis 1 and 2, which proposed a positive relationship between fit and employee well-being. To test Hypothesis 3, which predicted the mediation effect of work–family conflict on the relationship between P-E fit and employee well-being, bootstrap analysis was used.

Before testing our hypotheses, we examined the discriminant validity of our focal constructs by conducting confirmatory factor analyses (CFAs) to discern the discriminant validity among the seven latent variables. This factor analysis supported the discriminant validity of the measures (χ^2^ = 405.44, df = 215, *p* < 0.01; CFI = 0.94; TLI = 0.93; RMSEA = 0.05). We compared this model to one with all of the items loading on a single factor. Poor fit of this single-factor model indicating common method variance was not a significant problem within the data (χ^2^ = 1375.11, df = 230, *p* < 0.01; CFI = 0.65; TLI = 0.62; RMSEA = 0.11).

## 4. Results

Table 1 reports means, standard deviations, and correlations for all study variables. As shown in Table 1, boundary enactment is significantly positively correlated with all three dimensions of employee well-being (r = 0.22, *p* < 0.001; r = 0.45, *p* < 0.001; r = 0.42, *p* < 0.001) and significantly negatively correlated with work–family conflict (r = −0.50, *p* < 0.001). Furthermore, work–family conflict is significantly negatively correlated with all three dimensions of well-being (r = −0.36, *p* < 0.001; r = −0.53, *p* < 0.001; r = −0.49, *p* < 0.001).

### Hypothesis Testing

This study employed polynomial regression and response surface analysis for hypothesis testing. Following the recommendations of Shanock et al. (2010), the first step was to calculate the proportion of various pairings of the two focal variables within the sample to determine the necessity of polynomial regression analysis. The results showed that the proportion of cases where boundary preference matched actual boundary enactment was 31.7%; cases where boundary segmentation preference was less than actual boundary segmentation enactment accounted for 34.5%; and cases where boundary segmentation preference exceeded actual boundary segmentation enactment made up 33.8%. These findings confirmed the meaningfulness of using polynomial regression to study the matching problem.

**Figure 2:**
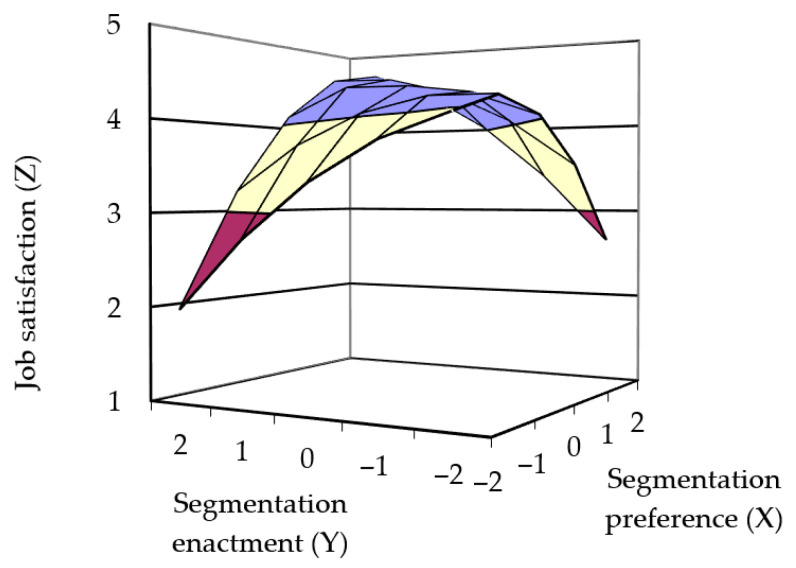
Three-dimensional surfaces depicting hypothesized relationship between P-E fit and job satisfaction.

Following Shanock et al. (2010), SPSS 25.0 was used to perform polynomial regression, and the results were utilized to create three-dimensional response surface plots. The polynomial regression results are shown in Table 2. Figure 2 illustrates the response surface for Model 1. The results of Model 1 indicated that the curvature of the regression along the incongruence line for boundary preference and actual boundary enactment on job satisfaction was significantly negative (curvature = −0.52, *p* = 0.003), suggesting that the more consistent boundary preference and actual boundary enactment were, the higher the job satisfaction. Thus, Hypothesis 1a was supported. The slope along the congruence line was not significant (slope = 0.19, *p* > 0.5; curvature = −0.01, *p* > 0.5), indicating no significant difference in job satisfaction between high and low levels of segmentation preference and actual segmentation when the two were consistent. Thus, Hypothesis 2a was supported.

**Figure 3:**
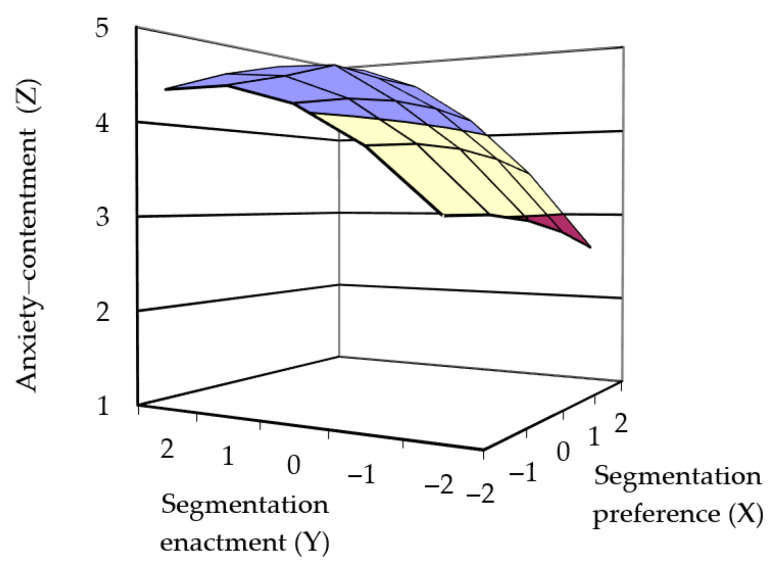
Three-dimensional surfaces depicting hypothesized relationship between P-E fit and anxiety–contentment.

Figure 3 depicts the response surface for Model 2. The results of Model 2 showed that the curvature of the response surface for job-related anxiety–comfort along the incongruence line was not significant (curvature = −0.21, *p* > 0.5), indicating no significant relationship between congruence and job-related anxiety–comfort. Thus, Hypothesis 1b was not supported. The slope along the congruence line was significantly positive (slope = 0.48, *p* = 0.006), suggesting that when boundary preference and actual boundary enactment were consistent, higher levels of actual segmentation were associated with higher job-related anxiety–comfort well-being. Therefore, Hypothesis 2b was supported.

**Figure 4:**
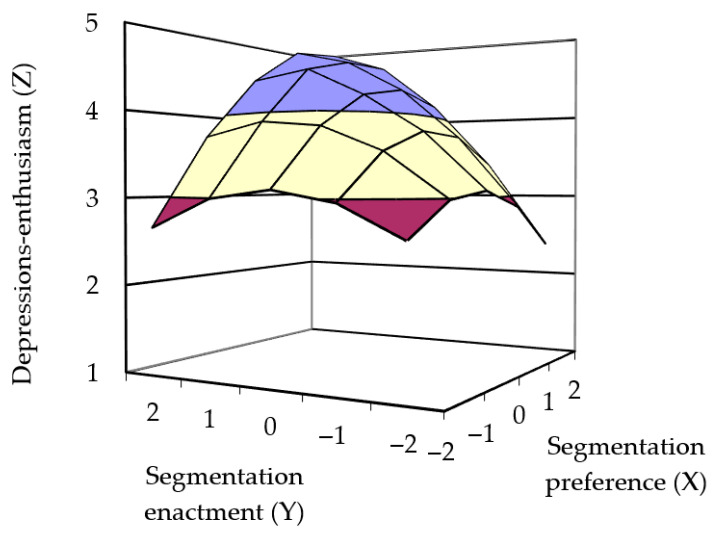
Three-dimensional surfaces depicting hypothesized relationship between P-E fit and depression–enthusiasm.

Figure 4 illustrates the response surface for Model 3. The polynomial regression results showed that the curvature of the response surface for job-related depression–enthusiasm along the incongruence line was significantly negative (curvature = −0.43, *p* = 0.009), indicating that greater consistency between boundary preference and actual boundary enactment led to higher job-related depression–enthusiasm well-being. Thus, Hypothesis 1c was supported. The slope along the congruence line was significantly positive (slope = 0.63, *p* = 0.001), suggesting that when boundary preference and actual boundary enactment were consistent, higher levels of actual segmentation were associated with higher job-related depression–enthusiasm well-being. Therefore, Hypothesis 2c was supported.

Hypothesis 3 predicts that work–family conflict mediates the relationship between boundary preference–actual boundary enactment congruence and employee well-being. Following the recommendations of Edwards and Cable (2009), this study employed the bootstrap method (with 10,000 resamples) to estimate the mediation effects. The results are shown in Table 3. Work–family conflict was significantly negatively correlated with job satisfaction (r = −0.33, *p* < 0.001), job-related anxiety–comfort (r = −0.40, *p* < 0.001), and job-related depression–enthusiasm (r = −0.40, *p* < 0.001). These findings indicate that higher levels of work–family conflict are associated with lower levels of all three dimensions of employee well-being. Work–family conflict mediated the relationship between boundary preference–actual boundary enactment congruence and employee well-being. Specifically, Boundary preference–actual boundary enactment congruence negatively influenced work–family conflict, which, in turn, positively impacted job satisfaction (effect = −0.33, 95% confidence interval [−0.47, −0.21]). It positively influenced job-related anxiety–comfort well-being (effect = −0.40, 95% confidence interval [−0.54, −0.27]). It positively influenced job-related depression–enthusiasm well-being (effect = −0.40, 95% confidence interval [−0.54, −0.27]). Thus, Hypotheses 3a, 3b, and 3c are supported.

## 5. Discussion

A survey of 420 employees from consulting firms revealed the following findings: (1) Consistency between work–family boundary preference and actual boundary enactment is positively correlated with job satisfaction and job-related depressive–enthusiastic well-being but has no significant relationship with job-related anxious–comfortable well-being. Although scholars in the work–family boundary field have begun to distinguish between individual boundary preferences and actual boundary enactment, prior research has typically focused on the effects of these variables in isolation on work–family outcomes, neglecting the impact of their alignment on employee well-being. These findings suggest that congruence, rather than the absolute direction or degree of boundary segmentation or integration, is more critical in supporting employees’ affective experience at work. This aligns with and extends recent findings emphasizing the importance of boundary management fit (Desrochers & Morgan, 2021; Michel et al., 2022; Reinke & Gerlach, 2022). (2) When boundary preference aligns with actual boundary enactment, there is no significant difference in job satisfaction whether both are high or low. This supports the idea that both integrators and segmenters can thrive when their work environment allows them to act in accordance with their personal preferences. However, compared to the “low segmentation preference–low segmentation enactment” category, job-related anxious–comfortable well-being and depressive–enthusiastic well-being are higher in the “high segmentation preference-high segmentation enactment” category. One possible explanation for this is that individuals with a high preference for segmentation may be more sensitive to role boundary violations and more deliberate in using boundaries to manage stress. When they successfully enact segmentation, they may experience a sense of control and psychological safety (Ashforth et al., 2000; Kossek et al., 2011), thus gaining more emotional benefits than integrators, who may adapt more flexibly but also face greater role overlap and ambiguity. (3) Work–family conflict mediates the relationship between the consistency of boundary preference and enactment and employee well-being (job satisfaction, job-related anxious–comfortable well-being, and job-related depressive–enthusiastic well-being). The mediating role of work–family conflict confirms role strain theory’s proposition that competing role demands are a key source of psychological tension (Kahn et al., 1964). Employees who experience misalignment between preferred and enacted boundaries likely encounter greater difficulty in managing role transitions, which increases work–family conflict and, in turn, impairs their well-being.

## 6. Conclusions

This study reaches the following conclusions: (1) The alignment between work–family boundary preferences and actual boundary enactment is positively correlated with job satisfaction and job-related depression–enthusiasm. (2) When boundary preferences align with actual boundary enactment, there is no significant difference in job satisfaction between high alignment and low alignment. However, compared to the “low segmentation preference–low segmentation enactment” category, job-related anxiety–comfort and job-related depression–enthusiasm are higher when the segmentation preference and segmentation enactment are both high. (3) Work–family conflict mediates the relationship between the alignment of boundary preferences and boundary enactment and employee well-being (job satisfaction, job-related anxiety–comfort, and job-related depression–enthusiasm). Alignment between boundary preferences and actual boundary enactment improves employee well-being by reducing work–family conflict.

### 6.1. Theoretical Implications

This study makes contributions in three key areas: Firstly, we offer a new perspective to explore the relationship between boundary management and employee well-being. Unlike the person–environment fit perspective, this study adopts an individual-centric approach to examine how the alignment between individual boundary preferences and actual boundary enactment affects employee well-being. Boundary theory (Ashforth et al., 2000; Clark, 2000) posits that individuals actively manage transitions between work and family domains using psychological, temporal, and spatial strategies. Our findings highlight that not only is the enactment of these boundaries important, but so too is their alignment with internal preferences—a nuance that extends boundary theory beyond structural or contextual considerations toward a more agentic, individualized perspective.

Secondly, previous studies have suggested that boundary preferences have no direct impact on work–family outcome variables, and conclusions regarding the positive or negative effects of actual boundary enactment on well-being have been inconsistent. Through empirical research, this study underscores the importance of individual boundary preferences, confirming that the alignment between boundary preferences and actual boundary enactment significantly influences employee well-being. This finding helps clarify inconsistencies in earlier conclusions, demonstrating that neither segmented nor integrated boundary enactment is inherently good or bad; rather, the key to well-being lies in whether actual boundary enactment aligns with individual preferences.

Thirdly, role theory (Kahn et al., 1964) argues that role conflict arises when demands from different domains are incompatible. The observed mediating role of work–family conflict in our study offers empirical support for this framework. Specifically, when boundary enactment violates internal preferences, it likely increases perceived role ambiguity or overload, which heightens inter-role conflict. This integration of boundary and role theory helps explain why boundary alignment enhances well-being: it enables smoother role transitions, reduces psychological strain, and supports cognitive clarity in navigating domain expectations.

### 6.2. Practical Implications

Although this study focused on the internal fit between boundary preference and enactment, it is important to recognize that such internal alignment often depends on the external environment (Ashforth et al., 2000). According to person–environment fit theory (Kristof-Brown et al., 2005), when organizational norms, expectations, or policies are congruent with individuals’ preferences, employees are more likely to enact boundaries in line with their values, thereby enhancing boundary fit and well-being. Therefore, organizations should create conditions that support a better alignment between employees’ boundary preferences and the organizational context. This includes not only fostering an environment conducive to diverse boundary management styles but also granting employees the autonomy to enact their preferred boundaries, which can in turn enhance motivation, engagement, and overall well-being.

Secondly, this study theoretically connects work–family boundary preference and the alignment of actual boundary enactment with employee well-being by proposing work–family conflict as a mediating mechanism, based on the idea that boundary management affects role management. By integrating boundary theory with role theory, our research contributes to advancing theoretical integration. Boundary theory focuses on transitions between roles, which are often constrained by space and time, relating to specific locations and specific times of the day or week. For example, employees are more likely to perform work roles in the workplace from Monday to Friday and family roles at home during evenings and weekends. When individuals cross the boundaries between work and family domains, their roles also transition. Thus, integrating boundary theory with role theory is necessary to explain the mechanism through which boundary alignment affects well-being.

Finally, this study explores the mediating role of work–family conflict in the relationship between boundary preference alignment and well-being. Work–family boundary issues span both the work and family domains. Introducing work–family conflict as a mediating mechanism based on role theory enhances our understanding of the relationship between boundary preference alignment and well-being. The empirical findings of this study confirm the explanatory power of work–family conflict as a mediating mechanism in this relationship. Although polynomial regression results indicate that boundary preference alignment does not directly influence related anxious–comfortable well-being, the mediating role of work–family conflict is significant, highlighting its importance in connecting boundary preference alignment and employee well-being.

Thus, this study extends research on the mediating mechanisms between boundary preference–enactment alignment and well-being by focusing on work–family conflict, providing a highly explanatory perspective for understanding this relationship. Organizations implementing policies to help employees reduce work–family conflict can further support the achievement of employee well-being.

### 6.3. Limitations and Future Directions

This study has several limitations: First, it is an exploratory investigation into the impact of the alignment between boundary preferences and actual boundary enactment on employee well-being. Allen et al. (2014) proposed that work and family boundary preferences and enactments involve physical, psychological, and behavioral dimensions, each emphasizing different aspects of boundaries. Physical boundaries focus on the location and time of performing tasks in a particular domain; psychological boundaries emphasize cognitive (thoughts and emotions) alignment with the domain an individual is in; behavioral boundaries highlight the consistency of social behavior patterns with the domain an individual occupies. The boundaries discussed in this study encompass all three dimensions as an overall concept. Future research could delve into individual dimensions to better understand the relationship between boundary preferences, actual boundary enactment, and well-being across these specific dimensions.

Second, work–family boundary issues are inherently bidirectional, encompassing both work-to-family and family-to-work domains. While this study centers on the work-to-family direction, future research should address the family-to-work direction, which poses distinct boundary management challenges. Notably, individuals may exhibit asymmetrical preferences and enactments across directions. Specifically, an individual may prefer to segment work from family, choosing not to respond to work emails or take calls at home (segmentation of work from family), while simultaneously integrating family life into work, for example, by displaying family photos in the office or occasionally attending to a child’s school matters during work hours (integration of family into work). Conversely, another person may integrate work into family life by working in the evenings or on weekends at home (integration of work into family life), yet they may keep their family out of the workplace by avoiding personal calls or discussions about family matters during work hours (segmentation of family from work). Future research could aim to examine bidirectional work–family boundary management.

Third, this study lacks consideration of contingency factors when exploring the mechanism through which boundary preference alignment affects well-being. Societal expectations for male and female roles differ, particularly in families with children requiring care. The enactment of work and family roles may vary by gender, and differences may also exist in boundary preferences and actual enactment. Furthermore, with a new generation of employees entering the workforce in large numbers, previous research has confirmed that these employees have unique personality traits and values, which might influence their perspectives and choices regarding work–family boundaries. Future research could investigate how boundary preferences and enactment influence well-being across different generations, genders, and family structures.

## Figures and Tables

**Table 1:** Descriptive statistics and correlations.

	Mean	SD	1	2	3	4	5
1. Boundary preference	4.13	0.54					
2. Boundary enactment	3.50	0.66	0.24 ***				
3. Job satisfaction	3.73	0.81	0.02	0.22 ***			
4. Anxiety–contentment	3.70	0.77	0.09	0.45 ***	0.40 ***		
5. Depression–enthusiasm	4.01	0.81	0.13 **	0.42 ***	0.45 ***	0.71 ***	
6. Work–family conflict	2.59	0.86	−0.13 **	−0.50 ***	−0.36 ***	−0.53 ***	−0.49 ***

Notes: N = 420. Cronbach’s alpha shown on primary diagonal. ** *p* < 0.01; *** *p* < 0.001.

**Table 2:** Results from polynomial regression of employee well-being on segmentation preference and enactment.

	Job Satisfaction	Anxiety–Contentment	Depression–Enthusiasm
Preference for segmentation	0.18	0.02	0.28
Segmentation enactment	0.01	0.45 **	0.35 *
Preference for segmentation Sqd.	−0.18 *	−0.04	−0.15
Preference × Enactment	0.25 *	0.07	0.17
Segmentation enactment Sqd.	−0.08	−0.11	−0.12
R^2^	0.12	0.23	0.21
ΔR^2^	0.05 ***	0.13 ***	0.14 ***
Linear shape along P = E (b1 + b2)	0.19	0.48 **	0.63 **
Curvilinear shape along P = E (b3 + b4 + b5)	−0.01	−0.08	−0.10
Linear shape along P = −E (b1 − b2)	0.17	−0.43 *	−0.07
Curvilinear shape along P = E (b3 − b4 + b5)	−0.52 **	−0.21	−0.43 **

Notes: N = 420. Unstandardized regression coefficients are reported. b1, b2, b3, b4, and b5 are the coefficients for preference for segmentation, segmentation enactment, preference for segmentation Sqd., preference × enactment, and segmentation enactment Sqd., respectively. Models also include the following control variables: gender, generation, marital status, parental responsibility, job status, hours worked, and life role priority. * *p* < 0.05; ** *p* < 0.01; *** *p* < 0.001.

**Table 3:** Analysis results regarding the impact of boundary segmentation preference–boundary enactment congruence on employee well-being via work–family conflict.

Variables	Job Satisfaction	Anxiety–Contentment	Depression–Enthusiasm
Mediating Effect	−0.33 (−0.47,−0.21)	−0.40 (−0.54, −0.27)	−0.40 (−0.55, −0.27)
Direct Effect	−0.12 (−0.35, 0.12)	−0.39 *** (−0.59, −0.19)	−0.42 *** (−0.64, −0.21)
Work–family Conflict	−0.33 *** (−0.43, −0.23)	−0.40 *** (−0.48, −0.31)	−0.40 *** (−0.50, −0.31)

*** *p* < 0.001.

## Data Availability

The data presented in this study are available on request from the corresponding author due to ethical reasons.
